# Air Pollution and the Risk of Cardiac Defects: A Population-Based Case-Control Study

**DOI:** 10.1097/MD.0000000000001883

**Published:** 2015-11-06

**Authors:** Bing-Fang Hwang, Yungling Leo Lee, Jouni J.K. Jaakkola

**Affiliations:** From the Department of Occupational Safety and Health, College of Public Health, China Medical University, Taichung (B-FH); Institute of Epidemiology and Preventive Medicine and Research Center for Genes, Environment and Human Health, College of Public Health, National Taiwan University, Taipei, Taiwan (YLL); and Center for Environmental and Respiratory Health Research, Institute of Health Sciences, University of Oulu, Oulu, Finland (JJKJ).

## Abstract

Previous epidemiologic studies have assessed the role of the exposure to ambient air pollution in the development of cardiac birth defects, but they have provided somewhat inconsistent results. To assess the associations between exposure to ambient air pollutants and the risk of cardiac defects, a population-based case-control study was conducted using 1087 cases of cardiac defects and a random sample of 10,870 controls from 1,533,748 Taiwanese newborns in 2001 to 2007.

Logistic regression was performed to calculate odds ratios for 10 ppb increases in O_3_ and 10 μg/m^3^ increases in PM_10_. In addition, we compared the risk of cardiac defects in 4 categories-high exposure (>75th percentile); medium exposure (75th to 50th percentile); low exposure (<50th–25th percentile); reference (<25th percentile) based on the distribution of each pollutant. The risks of ventricular septal defects (VSD), atrial septal defects (ASD), and patent ductus arteriosus (PDA) were associated with 10 ppb increases in O_3_ exposure during the first 3 gestational months among term and preterm babies. In comparison between high PM_10_ exposure and reference category, there were statistically significant elevations in the effect estimates of ASD for all and terms births. In addition, there was a negative or weak association between SO_2,_ NO_2_, CO, and cardiac defects_._

The study proved that exposure to outdoor air O_3_ and PM_10_ during the first trimester of gestation may increase the risk of VSD, ASD, and PDA.

## INTRODUCTION

Cardiac birth defects constitute the most common group of birth defects (∼50 per 1000 births),^1^ and the most common cardiac defects are ventricular septal defect (VSD) (27.5 /10,000 births), atrial septal defect (ASD) (10.6/10,000 births), and patent ductus arteriosus (PDA) (2.9/10,000 births).^2^ Epidemiologic studies have provided evidence of the possible effects of air pollutants on low birth weight, small gestational age, and preterm birth since 1990.^3–7^ In the past, only 12 epidemiologic studies elaborated the effects of exposure to ambient air pollution on the risk of cardiac defects during pregnancy,^8–19^ but these studies have provided inconsistent results. One meta-analysis suggested that NO_2_ and SO_2_ exposures were associated with coarctation of the aorta and tetralogy of Fallot, and PM_10_ exposure was associated with increased risk of ASDs.^20^ But the other one reported that only NO_2_ exposure was related to coarctation of the aorta.^21^ However, these studies did not adjust for maternal diabetes mellitus, smoking, and alcohol consumption during pregnancy, which are potential sources of confounding. In this study, a nationwide population-based case-control was conducted, and we collected the information on those important potential determinants for cardiac defects in pregnant women.

The exposure assessment in these studies was based on the measurement of monitoring stations nearest to the place of pregnancy during pregnancy, which may introduce exposure misclassification. Gilliland et al suggested that the exposure assessment should rely on the modeling approaches.^22^ Using a spatial modeling for exposure assessment, we elaborate the relations between women exposure to ambient air pollution during the first trimester and the risk of cardiac defects. We focused on predominantly traffic-related pollutants such as nitrogen dioxides (NO_2_), carbon monoxide (CO), and ozone (O_3_) and air pollutants mainly from other fossil fuel combustion sources, such as sulfur dioxide (SO_2_), and particles with an aerodynamic diameter of 10 μm or less (PM_10_).

## METHODS

### Study Design

We conducted a population-based case-control study of cardiac defects. The source population comprised of all 1,533,748 births registered by the Taiwanese Birth Registry from January 1, 2001, to December 31, 2007. We identified all the cases of cardiac defects without chromosomal defects in the source population during the study period. Birth records in the registry were sorted by the date of birth. Control subjects were selected randomly from the source population. The study protocol was approved by the Institutional Review Board of China Medical University, and it complied with the principles outlined in the Helsinki Declaration.

### Definition and Selection of Cases

All births delivered within 15 days are compulsorily reported to the Taiwan Birth Registration. Taiwanese pregnant women are 99% covered by national health insurance and access to prenatal care is free of charge and there are at least 10 visits during pregnancy. The follow-up time is from the 1st month after conception through 7 days after birth. Birth defects are mostly diagnosed by a physician, most often by a cardiologist. A validation study of the Taiwanese birth registration reported a low percentage of missing information (1.6%) and good validity (sensitivity and specificity was 92.8% and 99.6%, respectively) and reliability (Cohen's k statistics was 0.92) for preterm birth (<37 weeks of gestational age).^23^

We classified the cardiac defects into 6 categories which were similar with categories used by Gilboa and colleagues.^9^ The following categories of cardiac defects were applied: (1) VSDs not included in the conotruncal defects (n = 193); (2) ASDs (n = 194); (3) PDA (n = 213); (4) endocardial cushion defects (n = 23); (5) pulmonary artery and valve (n = 60); and (6) conotruncal defects (n = 404) including tetralogy of Fallot (n = 111), transposition of the great arteries (n = 70), truncus arteriosus communis (n = 60), double outlet right ventricle (n = 85), and aorticopulmonary window (n = 78). All cardiac defects were confirmed by autopsy, echocardiogram, or cardiac catheterization. The gestational age was counted from conception through date of birth using ultrasound. A total of 1087 subjects were identified with ample information on gestational age and air pollutants, and 17 cases from the mountainous region were excluded due to missing air pollution data from January 1, 2001, to December 31, 2007.

### Selection of Control Subjects

The control subjects were randomly selected from the source population. The eligibility criteria included: born during the study period; no birth defects present; and sufficient information on the gestational age and air pollutants. The case-control ratio was ∼1:10 to meet optimal statistical power. There are 10,870 controls in the final study population.

### Exposure Assessment

Ambient air monitoring data for sulfur dioxide (SO_2_), nitrogen dioxide (NO_2_), ozone (O_3_), carbon monoxide (CO), and particles with an aerodynamic diameter of 10 μm or less (PM_10_) are available for 72 EPA monitoring stations on Taiwan's main island since 1994. Concentrations of each pollutant are measured continuously and reported hourly—CO by nondispersive infrared absorption, NO_2_ by chemiluminescence, O_3_ by ultraviolet absorption, SO_2_ by ultraviolet fluorescence, and PM_10_ by beta-gauge.

We identified the map coordinates of the monitoring stations and air pollution sources. The data were managed by a geographic information system (GIS) (ArcGIS 10.0). The air pollutant measurements from EPA monitoring stations were integrated into monthly point data and interpolated to pollutant surfaces using the inverse distance weighting (IDW) method. The monitoring data was assigned to women individually by a zip code. Zip codes typically stand for one block in urban areas (17.00 square kilometer, SD: 8.56) but in rural areas they correspond to larger (154.00 square kilometer, SD: 104.39) districts with lower population density. This method provided high temporal resolution (daily measures for most days) and suitable spatial resolution (100 m). We assigned for each day a concentration from 3 closest monitoring stations within 25 km. We then computed the monthly mean average for each woman during pregnancy. The details of the approach are described elsewhere.^24^ The air pollutant information for each woman during pregnancy, corresponding to the zip-code level residence, was extracted from the derived concentration surface maps using ArcGIS Spatial Analyst tool (developed by ESRI).

Exposure parameters were calculated from the monthly 24-h NO_2_, CO, SO_2_, PM_10_, and 8-h O_3_ average concentrations for the duration of pregnancies between 2000 and 2007. Based on the date of birth and gestational age, we estimated the monthly average concentration corresponding to the first trimester of gestation.

### Covariates

The following covariates were available from routine birth registration: sex of infant (male; female), maternal age (<20 years; 20–34 years; > = 35 years), plurality (singleton; multiple birth), gestational age (weeks), maternal smoking, alcoholic habit and medication during pregnancy, season of conception (spring; summer; fall; winter), and maternal health status defined as the presence of any of the following diseases or conditions: diabetes mellitus, anemia (HCT < 0.30/HGB < 0.10), cardiac disease, acute or chronic lung disease, genital herpes, hydraminios/oligohydramnios, chronic hypertension, pregnancy-associated hypertension, eclampsia, imcompetent cervix, renal disease, Rh sensitization, uterine bleeding (yes; no). The municipal level data was collected from the Department of Household Registration Affairs, Taiwanese Population Data Services, which were used to construct municipal level population density, which is a measure of the proportion of urban population in the municipality. A census-based socioeconomic status (SES) was derived from the 2005 national health insurance survey of the average monthly income of ∼9,700,000 households. All subjects were assigned an SES value, according to their place in residence. All average monthly incomes of households were standardized using Z scores following normal distribution N (μ = 0,σ^2^ = 1). SES quintiles were determined from the distribution and assigned to their appropriate quintile: quintile 1 containing the most affluent wards and quintile 5 the most deprived.

### Statistical Methods

We focused on the first 3 months (first trimester) of pregnancy, because the relevant embryologic period for cardiac defects is between the 4th and 12th week of gestation.^25^ We used odds ratio (OR) as a measure of the association between exposure to air pollution and the risk of cardiac defects. We performed logistic regression analysis to adjust for possible confounding factors. The goodness of fit was assessed with likelihood ratio tests to determine whether a variable contributed significantly to the model. First, we fitted a full model with a complete set of covariates. To elaborate sources of confounding, we fitted models with different combinations of covariates and compared the effect from models with and without the covariate of interest. If the removal of a covariate changed the studied effect estimate >10%, the corresponding covariate was kept in the final model.^26^ We first fitted 1-pollutant models, and then considered 2-pollutant models by fitting 1 traffic-related and 1 stationary fossil fuel combustion-related pollutant. Finally, we fitted 2-pollutant models with O_3_ and another pollutant (CO, NO_2_, and SO_2_). It was not appropriate fit 2-pollutant models with O_3_ and PM_10_ because of high collinearity (correlation coefficient *r* = 0.54). The 2-pollutant models provide estimates of the independent effects of CO, NO_2_, SO_2_, PM_10_, and O_3_ on cardiac defects controlling for the second pollutant in the model. The effect of each pollutant on the risk of cardiac defects was presented as ORs per 10 ppb changes for NO_2_, and O_3_, 100 ppb changes for CO, 10 μg/m^3^ changes for PM_10_, and 1 ppb for SO_2_, along with their 95% confidence intervals (CIs). We also compared the risk of cardiac defects in 4 exposure categories based on the distribution of each pollutant representing high (>75th percentile), medium (75th to 50th percentile), low exposure (<50th to 25th percentile), and < 25th percentile as the reference category. Because PDA as a congenital malformation is usually only diagnosed in term infants and only after the first few days of life, it may be better to assess PDA by term births only. We further performed sensitivity analyses by comparing the effect estimates between all and term births (gestational age >37 weeks).

## RESULTS

### Characteristics of Control and Case Subjects

A larger proportion of cases than controls had older mothers, maternal diabetes mellitus, lower SES, and shorter gestational age, and were from multiple births (Table 1). We adjusted for these factors in the multivariate analysis.

**TABLE 1:**
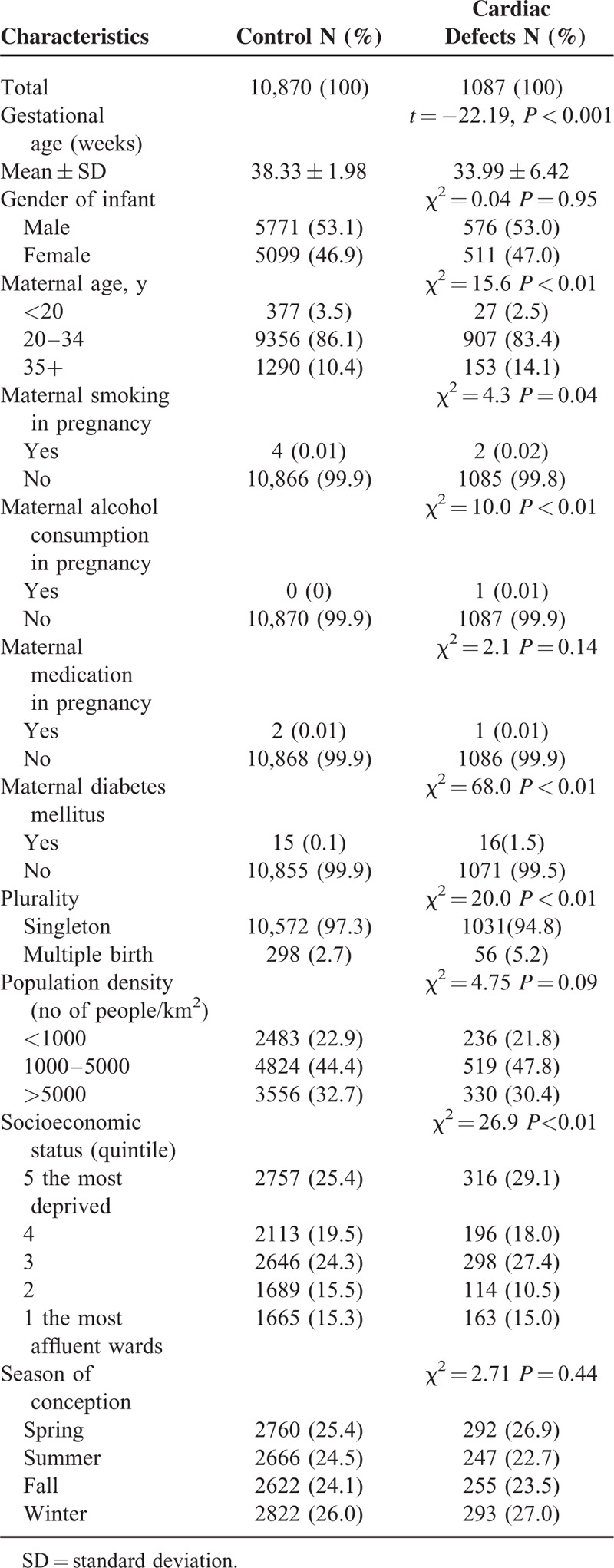
Characteristics of Controls and Cases of Cardiac Defects in Taiwan, 2001 to 2007

### Air Pollution

The distributions of the monthly mean air pollutant concentrations in different seasons from 72 monitoring stations in Taiwan 2001 to 2007 are shown in Table 2. The association between NO_2_ and CO trimester average concentrations during the first trimester was high (*r* = 0.80), which represent the common source of motor vehicles. The concentrations of PM_10_ and SO_2_ were also highly correlated (*r* = 0.53) indicating a common source of stationary fuel combustion, although SO_2_ concentrations were also associated with both traffic-related pollutants. The concentrations of O_3_ were moderately associated with PM_10_ (*r* = 0.54), and positively but weakly correlated with SO_2_ (*r* = 0.18). O_3_ was negatively correlated with the mainly traffic-related pollutants (Table S1, http://links.lww.com/MD/A486).

**TABLE 2:**
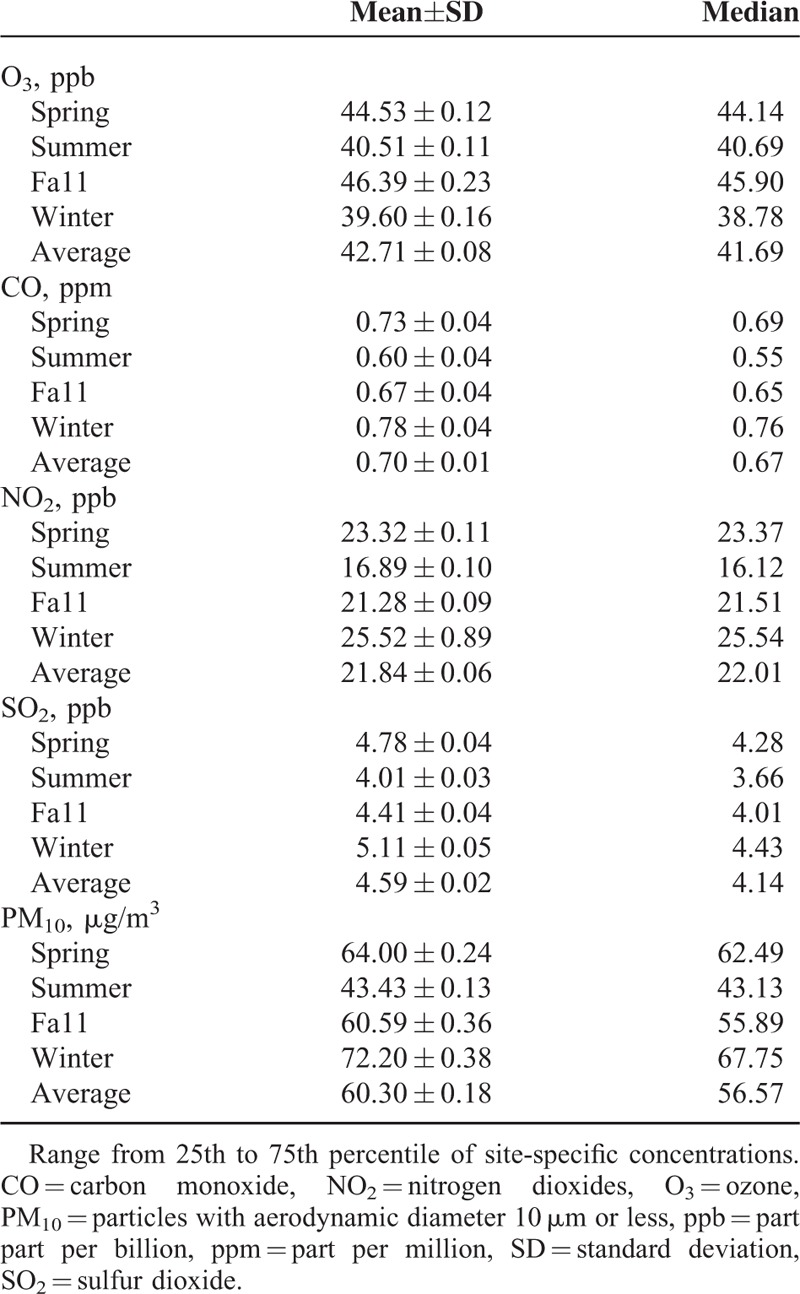
Mean and Distribution of Air Pollutions in Different Seasons Form in Taiwan 2001 to 2007

### Air Pollution and the Risk of Ventricular Septal Defects

The adjusted ORs for 10 ppb change in O_3_ for VSDs in the single-pollutant model were 1.31 (95% CI: 1.10–1.57) among all births and 1.49 (95% CI: 1.20–1.85) among term births for the first trimester of pregnancy, respectively (Tables 3 and 5). Similar ORs were found in the 2-pollutant models and the estimates increased a little when added different second pollutants (Table 4). Comparing the adjusted OR for medium and high O_3_ exposure to low exposure, the risk of VSDs was significantly increased (adjusted OR_medium_ O_3_ = 2.53, 95% CI: 1.55–4.14; adjusted OR_high_ O_3_ = 2.34, 95% CI: 1.41–3.90) in the single pollutant model. Furthermore, inclusion of both of the traffic-related pollutants (CO or NO_2_) and stationary fossil fuel combustion-related air pollutants (SO_2_) increased the effect estimate a little (Table 4). We did not find any association between other air pollutants and the risk of VSDs.

**TABLE 3:**
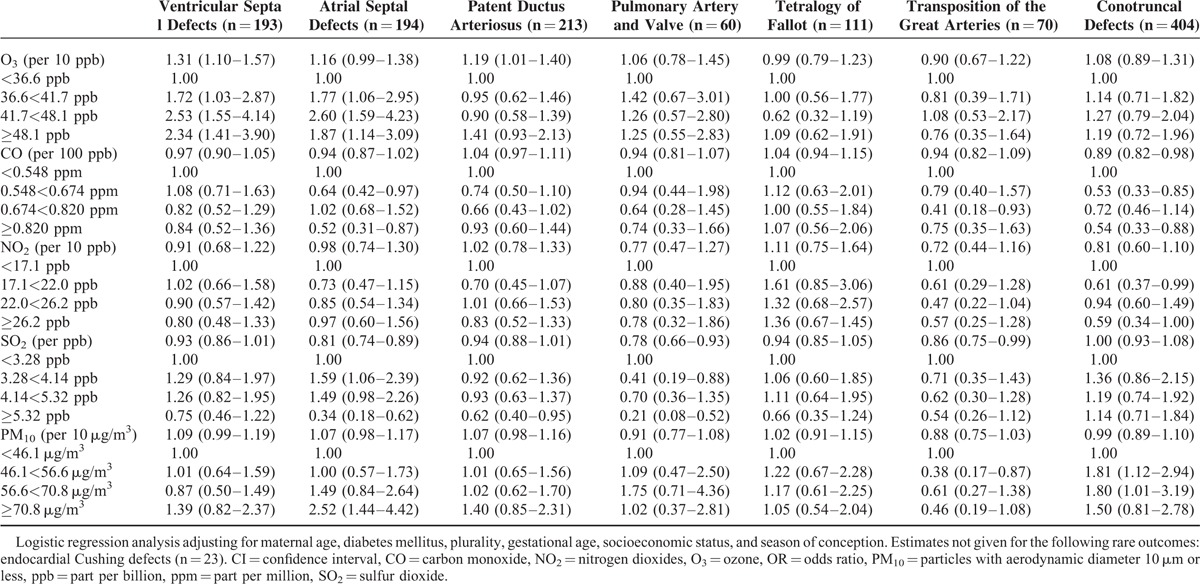
Adjusted Odds Ratios^∗^ (ORs), Along With 95% Confidence Interval (CIs) of Cardiac Defects Among All Births by Average Concentration and Quartile During the First Trimester of Pregnancy in Single Model

**TABLE 4:**
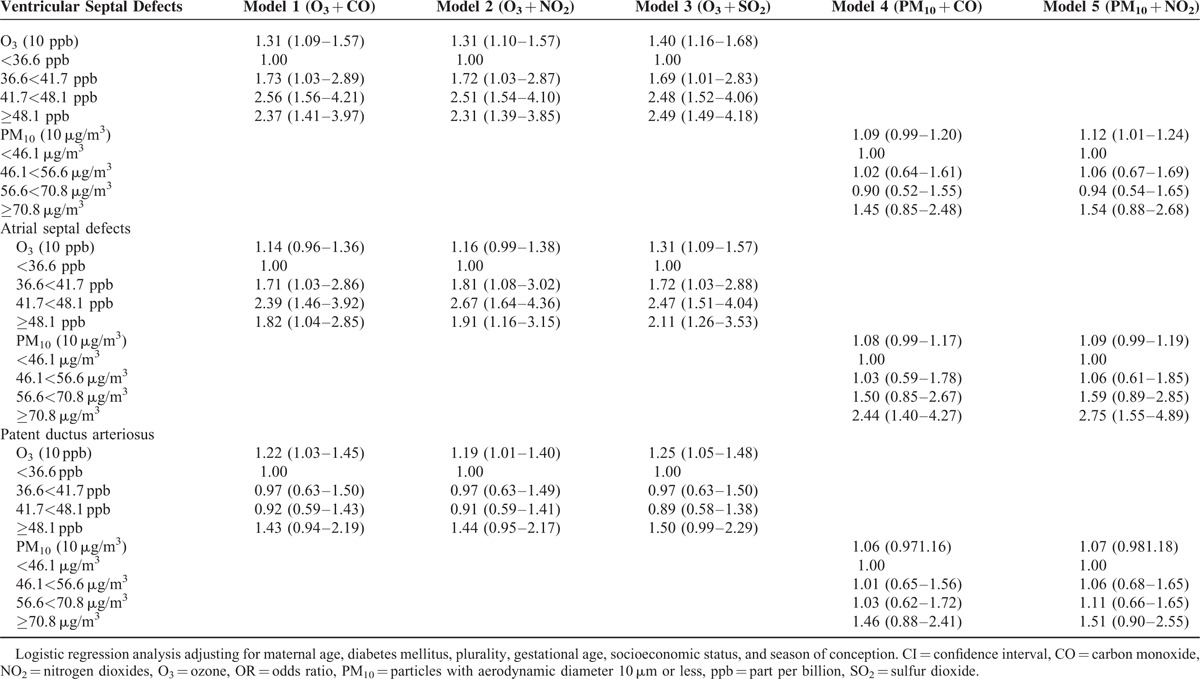
Adjusted Odds Ratios^∗^ (ORs), Along With 95% Confidence Interval (CIs) of Cardiac Defects, Ventricular Septal Defects, Atrial Septal Defects, and Patent Ductus Arteriosus Among All Births by Average Concentration and Quartile During the First 3 Months of Pregnancy in 2-Pollutant Models

### Air Pollution and the Risk of Atrial Septal Defects

The effect estimates for ASDs were elevated in the first trimester for all births (adjusted OR = 1.16, 95% CI: 0.99–1.38)_,_ but not statistically increased for term births (adjusted OR = 1.15, 95% CI: 0.94–1.41) for 10 ppb change in O_3_ (Tables 3 and 5).

**TABLE 5:**
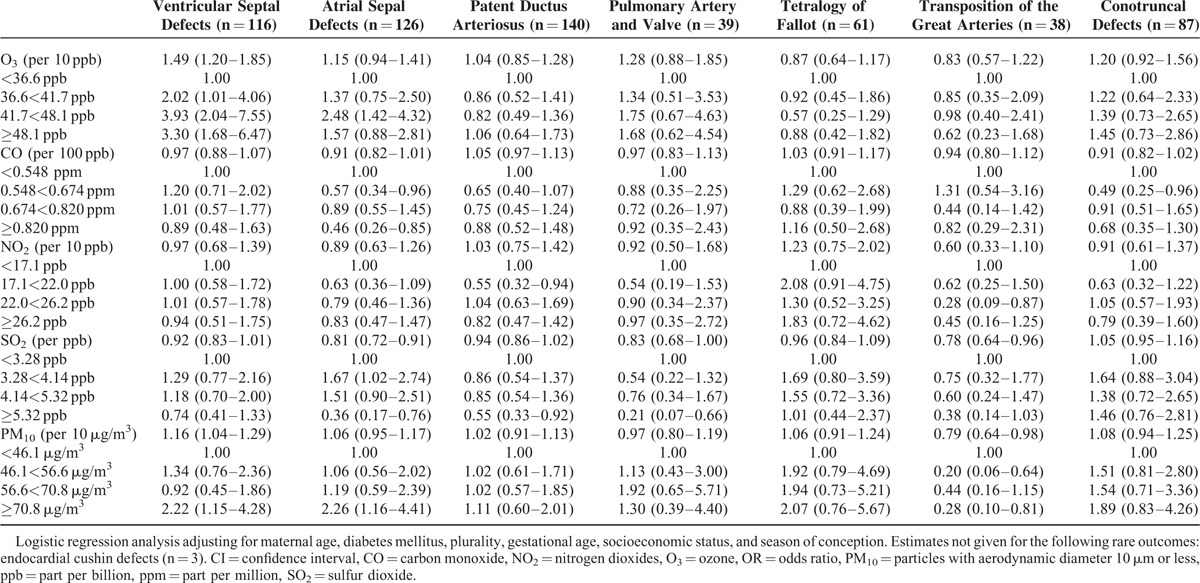
Adjusted Odds Ratios^∗^ (ORs), Along With 95% Confidence Interval (CIs) of Cardiac Defects Focusing on Term Births by Average Concentration and Quartile During the First Trimester of Pregnancy in Single Model

The risk of ASDs was also associated with 10 μg/m^3^ change in PM_10_ in the first trimester of pregnancy (adjusted OR = 1.07, 95% CI: 0.98–1.17) for all births, and inclusion of both of the traffic-related pollutants and O_3_ did not change the effect estimate substantially (Table 4). The effect estimates for ASDs for all births with high PM_10_ exposures were statistically elevated as compared to low exposures (adjusted OR_high_ PM_10_ = 2.52, 95% CI: 1.44–4.42) (Table 3). When focusing on term births, the effect estimates were also significantly increased comparing high PM_10_ exposures to low exposures (adjusted OR_high_ PM_10_ = 2.26, 95% CI: 1.16–4.41) (Table 5).

### Air Pollution and the Risk of Patent Ductus Arteriosus

The risk of PDA was related to 10 ppb O_3_ changes in first 3 months gestation (adjusted OR = 1.19, 95% CI: 1.01–1.40) for all births, but not for term births (adjusted OR = 1.04, 95% CI: 0.85–1.28) in the single-pollutant model (Tables 3 and 5). The effect estimates for PDA were increased, but not statistically significant in high O_3_ exposure (adjusted OR = 1.41, 95% CI: 0.93–2.13) in single-pollutant model, but inclusion of combustion-related pollutant SO_2_ changed the effect estimate a little (Table 4). The adjusted OR for 10 μg/m^3^ change in PM_10_ for PDA for all births in the single-pollutant model was 1.07 (95% CI: 0.98–1.16), but did not show statistical significance for term births (adjusted OR = 1.02, 95% CI: 0.91–1.13) (Table 5).

In summary, the risks of VSDs and ASDs for overall and term births were elevated with the continuous O_3_ exposure, but the risk of PDA was increased only for all births. The effect estimates of ASDs for the first trimester with continuous and categorical PM_10_ exposure was significantly increased when compared high exposures to low exposures for all and term births. Surprisingly, an inverse association between SO_2_ exposure and cardiac defects, particular in VSDs, ASDs, transposition of the great arteries was found. There were weak or no associations between other air pollutants and pulmonary artery and valve, tetralogy of Fallot, transposition of the great arteries, and conotruncal defects.

## DISCUSSION

### Principal Findings

The results provide evidence that O_3_ and PM_10_ exposure during the first trimester of gestation may increase the risk of VSD, ASD, and PDA. In addition, there was a negative or weak association between SO_2_, NO_2_, CO, and cardiac defects.

### Comparison With Other Studies

Twelve previous studies, conducted in Southern California,^8^ San Joaquin Valley of California,^15^ Texas,^9,19^ Atlanta,^10^ Australia,^11^ and United Kingdom,^12–14^ Israeli,^16^ Barcelona,^18^ and NBDPS (National Birth Defects Prevention Study) in 9 US States, ^17^ have investigated associations between cardiac defects and exposure to ambient air pollution. The present study found that per 10 ppb increase in O_3_ exposure during the first 3 months of gestation among all births were associated with the increased risk of VSDs (31%), ASDs (16%), and PDA (19%) respectively. The monthly average of O_3_ varied from 13.8 ppb to 86.3 ppb. This is different from the results of the Southern Californian study,^8^ which reported a CO exposure-related increase response (OR_low_ = 1.62, 95% CI: 1.05, 2.48; OR_medium_ = 2.09, 95% CI: 1.19, 3.67; OR_high_ = 2.95, 95% CI: 1.44, 6.05) and the Texan study,^9^ which showed an association between SO_2_ and VSDs (OR_high_ = 2.16, 95% CI: 1.51, 3.09). Other studies in Atlanta, Australia, United Kingdom, San Joaquin Valley of California, Israeli, Barcelona, and NBDPS reported no other or inverse associations between the criteria pollutant levels and VSDs.^12–18^ Our study indicated that PM_10_ exposure during the first trimester of gestation has increased risk of ASDs (OR_high_ = 2.52, 95% CI = 1.44, 4.42). Similar results were reported from the Texan study^9^ for ASDs (OR_high_ = 2.27, 95% CI = 1.43, 3.60) when high (>75th percentile) was compared with < 25th percentile as the reference category and Atlanta study for PDA (adjusted OR = 1.60 per 14.2 μg/m^3^ 95% CI 1.11, 2.31),^10^ but inconsistent results were found in San Joaquin Valley of California, Israeli, Barcelona, NBDPS, and Texas.^16–19^ The other 2 studies conducted in northeastern England show weak associations between black smoke and cardiac defects (adjusted OR = 1.02 per 1 μg/m^3^ 95% CI 1.01, 1.03), but they did not find a positive association for PDA.^12,14^ In our study, the risk of PDA was related to O_3_ exposure in first 3 months gestation for all births, but not for term births. These differences in effect estimates between all births and term births could be explained by information bias related to greater use of ultrasound in term births than in preterm births.

### Strengths and Limitations of Study

The strengths of our study include a comprehensive population-based case-control design (all the births in Taiwan), the ability to collect air pollution data from numerous places around the island corresponding to residence of women during pregnancy, and control important risk factors of cardiac defects, such as maternal diabetes mellitus. Our outcomes of interest were based on birth registration rather than the clinical examination for the purposes of the study. The cardiac defects might be missed or underreported in Taiwan, because we only include the defects diagnosed up to 7 days of age (1.47/1000 births),^27^ compared with the Atlanta 1998 to 2005 reported rates (8.14/1000 births), which were diagnosis through 1 year of age. Our case ascertainment taking place during the first week of life may have introduced both random and systematic error leading to both over diagnosis and under diagnosis. For example, the presence of a PDA in the first week of life does not reflect a true congenital anomaly but a neonatal finding that may be normal. Similarly a diagnosis of an atrial defect in this period may be an over reading of a patent foramen ovale or clinically insignificant small atrial defect. This is a possible source of misclassification, which is likely to be random and nondifferential between women exposure to high and low levels of ambient air pollution and thus likely to lead to underestimation of the effect estimates. Although this would depend on whether tertiary care hospitals which might diagnosis more defects are located in the densely populated areas where pollution levels would be higher, we did not find areas of greater pollution in Taiwan. The echocardiograms are commonly performed on infants in Taiwan and the prevalence of certain cardiac defects did not show substantially differences over the study period. In our study, the percentage of premature births was higher among cases than the controls. Even though gestational age (weeks) in the multivariate analysis was adjusted for the potential differences between cases and controls, we still cannot rule out the either a consequence of cardiac defects or a common cause shared between the defects and premature birth.

Because of low occurrence of maternal smoking, alcohol consumption, and medication during pregnancy between case and control groups, it is not meaningful to adjust for these factors (Table 1). It is not possible to take some confounders such as occupational exposure, maternal work or travel, vitamin use, diet, and folic acid into consideration,^28^ because there was no such information available in Taiwanese birth registration data. As these factors may have seasonal and regional variations, we included season of conception and population density to adjust indirectly not only for these factors, but also municipal differences in these behavior factors. However, potential residual confounding might be unmeasured or poorly characterized by other environmental toxicants.

The differences between personal exposure and municipal level exposure could be explained by known or unknown factors such as behavior pattern, living activity, working history, and indoor air pollution. Nondifferential errors were assumed between cases and controls. The present and all the previous studies on cardiac defects are adjusted only for covariates based on birth registration information.^8–14^ Our nationwide population-based case-control study based on Taiwanese birth registration has the advantage of having larger numbers of births which would reduce the uncertainly due to the random error typical for smaller studies that collected detailed information on covariates from pregnant women.^29^

Our exposure assessment was based on the residential zip code rather than on the address during pregnancy, and we applied GIS to integrate monthly air pollutant data from 72 EPA monitoring stations which was interpolated to pollutant surfaces using the IDW method. Two previous studies reported that when using the municipal level exposure obtained from air pollution monitoring stations as a proxy for personal exposure results in smaller effect estimates than when using personal assessment of exposure.^30,31^ A plausible explanation of information bias is residential mobility during pregnancy may lead to exposure misclassification. Any random migration in cases and controls might introduce nondifferential misclassification and decrease the accuracy of exposure assessment. This would most likely result in underestimation of the air pollution effects rather than a positive bias in the associations.

Since urban air pollution usually consists of a complex mixture of several compounds, evaluating the independent effects of different pollutants and identifying a candidate teratogen is not easy. The results of Pearson's correlation analysis during the first trimester of pregnancy showed a high correlation (*r* = 0.80) between NO_2_ and CO, since they are both emitted predominantly from motor vehicles. Likewise there exited a moderate correlation (*r* = 0.53) between PM10 and SO_2_ with important sources from stationary combustion of fossil fuels. O_3_ is a secondary air pollutant produced in the lower atmosphere from precursors of the vehicle emissions (nitrogen dioxide and hydrogen carbon), but the concentrations of O_3_ are highly associated with PM_10_ (*r* = 0.54) and slightly related to NO_2_ (*r* = −0.07), CO (*r* = −0.27), and SO_2_ (*r* = 0.18) concentrations. To some degree, this correlation analysis enables us to validly evaluate the effects of O_3_ on cardiac defects independent from NO_2_, CO, and SO_2_. Meanwhile it is possible to control 1 potential confounder (stationary fossil fuel pollutant) at a time in evaluating the effect of different traffic-related pollutants.

This study investigated a relatively large number of health outcomes, which may influence the interpretation of the results. Since the hypotheses of the effects on *a priori* defined cardiac defect groups are independent and mutually exclusive, multiple-inference procedures were no longer required.^32^ According to Greenland and Rothman (1998),^33^ all the single-inference procedures with point estimates and CIs were presented in this study. However, selected effect estimates from an unknown number of estimates were not presented. Given 40 associations (8 outcomes × 5 air pollutants) present here, we would expect at least 2of the associations to be significant due to chance (if α = 0.05). Although our findings suggest that the risk of several cardiac defects is related to exposure to O_3_ and PM_10_ in time windows that match with our knowledge about susceptible periods of cardiac development, we cannot rule out the possibility of chance.

### Possible Mechanisms

How pregnant women's exposure to airborne particulate matter induces development of cardiac defects is still unknown and needs further investigation. The possible explanation is that aromatic hydrocarbons (PAHs) and heavy metals associated with inhaled particulate may cause DNA damage in male germ cells and changes in humans during development.^34,35^ An animal study revealed that high exposure to O_3_ (>1.26 ppm) during organogenesis had embryocidal effects in rats.^36^ As we know, vitamin A deprivation during organogenesis causes several congenital defects, rats exposed to 0.4 ppm O_3_ for 1 to 4 days had an 85% lowering of the serum retinol concentration,^37,38^ supporting the hypothesized adverse effects of O_3_. Exposure to O_3_ was associated with the e risk of cardiac defects. The most susceptible time to the effects of O_3_ were the first 3 months of gestation. O_3_ is considered to be a strong oxidizing agent to generate hydrogen peroxide, hydroxyl radicals, and super oxides. It was related to oxidative stress and the development of cardiac defects.

Our finding of lack of association between the risk of cardiac defects and traffic-related (CO and NO_2_) and combustion-related (SO_2_) air pollutant levels is consistent with the results from Atlanta,^10^ Australia,^11^ and United Kingdom.^12–14^

## CONCLUSIONS

The present study provides evidence that the effect of exposure to outdoor air O_3_ and PM_10_ during the first 3 months of pregnancy increases the risk of cardiac defects. Given that similar levels are encountered globally by large numbers of pregnant women, O_3_ and PM_10_ may be an important determinant of cardiac defects.

## UNCITED REFERENCES

^31–38^.
